# Supplementary material for: Non-Pharmacological Therapies for Post-Viral Syndromes, Including Long COVID: A Systematic Review
Source: Int J Environ Res Public Health. 2023 Feb 16;20(4):3477. doi: 10.3390/ijerph20043477 (PMC9967466; doi:10.3390/ijerph20043477)
Supplement: Supplementary file 1 [file ijerph-20-03477-s001.zip › ijerph-2184243-supplementary.pdf]

Table S1 – PRISMA checklist

Section and Topic	Item #	Checklist item	Location where item is reported
**TITLE**			
Title	1	Identify the report as a systematic review.	1
**ABSTRACT**			
Abstract	2	See the PRISMA 2020 for Abstracts checklist.	3
**INTRODUCTION**			
Rationale	3	Describe the rationale for the review in the context of existing knowledge.	5
Objectives	4	Provide an explicit statement of the objective(s) or question(s) the review addresses.	5
**METHODS**			
Eligibility criteria	5	Specify the inclusion and exclusion criteria for the review and how studies were grouped for the syntheses.	6
Information sources	6	Specify all databases, registers, websites, organisations, reference lists and other sources searched or consulted to identify studies. Specify the date when each source was last searched or consulted.	6
Search strategy	7	Present the full search strategies for all databases, registers and websites, including any filters and limits used.	6
Selection process	8	Specify the methods used to decide whether a study met the inclusion criteria of the review, including how many reviewers screened each record and each report retrieved, whether they worked independently, and if applicable, details of automation tools used in the process.	7
Data collection process	9	Specify the methods used to collect data from reports, including how many reviewers collected data from each report, whether they worked independently, any processes for obtaining or confirming data from study investigators, and if applicable, details of automation tools used in the process.	7
Data items	10a	List and define all outcomes for which data were sought. Specify whether all results that were compatible with each outcome domain in each study were sought (e.g. for all measures, time points, analyses), and if not, the methods used to decide which results to collect.	7
	10b	List and define all other variables for which data were sought (e.g. participant and intervention characteristics, funding sources). Describe any assumptions made about any missing or unclear information.	7
Study risk of bias assessment	11	Specify the methods used to assess risk of bias in the included studies, including details of the tool(s) used, how many reviewers assessed each study and whether they worked independently, and if applicable, details of automation tools used in the process.	7
Effect measures	12	Specify for each outcome the effect measure(s) (e.g. risk ratio, mean difference) used in the synthesis or presentation of results.	7
Synthesis methods	13a	Describe the processes used to decide which studies were eligible for each synthesis (e.g. tabulating the study intervention characteristics and comparing against the planned groups for each synthesis (item #5)).	7
	13b	Describe any methods required to prepare the data for presentation or synthesis, such as handling of missing summary statistics, or data conversions.	7

Section and Topic	Item #	Checklist item	Location where item is reported
	13c	Describe any methods used to tabulate or visually display results of individual studies and syntheses.	7
	13d	Describe any methods used to synthesize results and provide a rationale for the choice(s). If meta-analysis was performed, describe the model(s), method(s) to identify the presence and extent of statistical heterogeneity, and software package(s) used.	7
	13e	Describe any methods used to explore possible causes of heterogeneity among study results (e.g. subgroup analysis, meta-regression).	7
	13f	Describe any sensitivity analyses conducted to assess robustness of the synthesized results.	7
Reporting bias assessment	14	Describe any methods used to assess risk of bias due to missing results in a synthesis (arising from reporting biases).	7
Certainty assessment	15	Describe any methods used to assess certainty (or confidence) in the body of evidence for an outcome.	7
**RESULTS**			
Study selection	16a	Describe the results of the search and selection process, from the number of records identified in the search to the number of studies included in the review, ideally using a flow diagram.	9
	16b	Cite studies that might appear to meet the inclusion criteria, but which were excluded, and explain why they were excluded.	8
Study characteristics	17	Cite each included study and present its characteristics.	9-12
Risk of bias in studies	18	Present assessments of risk of bias for each included study.	13
Results of individual studies	19	For all outcomes, present, for each study: (a) summary statistics for each group (where appropriate) and (b) an effect estimate and its precision (e.g. confidence/credible interval), ideally using structured tables or plots.	14-16
Results of syntheses	20a	For each synthesis, briefly summarise the characteristics and risk of bias among contributing studies.	10-16
	20b	Present results of all statistical syntheses conducted. If meta-analysis was done, present for each the summary estimate and its precision (e.g. confidence/credible interval) and measures of statistical heterogeneity. If comparing groups, describe the direction of the effect.	No statistics undertaken
	20c	Present results of all investigations of possible causes of heterogeneity among study results.	No statistics undertaken
	20d	Present results of all sensitivity analyses conducted to assess the robustness of the synthesized results.	No statistics undertaken
Reporting biases	21	Present assessments of risk of bias due to missing results (arising from reporting biases) for each synthesis assessed.	N/A
Certainty of evidence	22	Present assessments of certainty (or confidence) in the body of evidence for each outcome assessed.	N/A
**DISCUSSION**			

Section and Topic	Item #	Checklist item	Location where item is reported
Discussion	23a	Provide a general interpretation of the results in the context of other evidence.	19
	23b	Discuss any limitations of the evidence included in the review.	19-21
	23c	Discuss any limitations of the review processes used.	21
	23d	Discuss implications of the results for practice, policy, and future research.	19-21
**OTHER INFORMATION**			
Registration and protocol	24a	Provide registration information for the review, including register name and registration number, or state that the review was not registered.	3
	24b	Indicate where the review protocol can be accessed, or state that a protocol was not prepared.	5
	24c	Describe and explain any amendments to information provided at registration or in the protocol.	6 and 20
Support	25	Describe sources of financial or non-financial support for the review, and the role of the funders or sponsors in the review.	2
Competing interests	26	Declare any competing interests of review authors.	2
Availability of data, code and other materials	27	Report which of the following are publicly available and where they can be found: template data collection forms; data extracted from included studies; data used for all analyses; analytic code; any other materials used in the review.	N/A

Figure S1 – Search strategy from MEDLINE

Search Strategy:

#	Searches	Results
1	("myalgic encephalomyelitis" or postviral or "post viral" or "post infectious" or postinfectious or recurr* or reoccur* or chronic or persistent or longterm or "long term" or longhaul or "long haul").mp.	3039608
2	"FATIGUE SYNDROME, CHRONIC"/	5785
3	1 or 2	3039608
4	(ebolavirus or "middle eastern respiratory" or "severe acute respiratory" or MERS or SARS or ebola or marburg or "epstein barr" or "human herpesvirus" or cytomegalovirus or "varicella zoster" or "herpes simplex" or influenza or coxsackie or coxsackievirus or "hepatitis A" or enterovirus* or rhinovirus* or parvovirus* or "ross river" or chikungunya or "semliki forest" or "west nile" or dengue or "yellow fever" or zika or "mayaro virus" or rotavirus* or mumps or measles or parainfluenza or "respiratory syncytial" or "human metapneumovirus" or adenovirus* or norovirus* or "vomiting bug" or sapovirus* or astrovirus* or coronavirus or SARS-CoV-2 or novelcovid or 2019-nCoV or novelcoronavirus or covid19 or covid).mp.	681904
5	CORONAVIRUS/ or NOROVIRUS/ or SAPOVIRUS/ or exp *ADENOVIRIDAE/ or METAPNEUMOVIRUS/ or "PARAINFLUENZA VIRUS 1, HUMAN"/ or "PARAINFLUENZA VIRUS 3, HUMAN"/ or "PARAINFLUENZA VIRUS 2, HUMAN"/ or "PARAINFLUENZA VIRUS 4, HUMAN".mp. or "RESPIRATORY SYNCYTIAL VIRUS, HUMAN"/ or MEASLES/ or MUMPS/ or ROTAVIRUS/ or "ZIKA VIRUS"/ or "YELLOW FEVER VIRUS"/ or exp *DENGUE/ or "SEMLIKI FOREST VIRUS"/ or "WEST NILE VIRUS"/ or "ROSS RIVER VIRUS"/ or "CHIKUNGUNYA VIRUS"/ or PARVOVIRUS/ or "PARVOVIRUS B19, HUMAN"/ or RHINOVIRUS/ or "HEPATITIS A"/ or "INFLUENZA, HUMAN"/ or exp *ENTEROVIRUS/ or "HERPES SIMPLEX"/ or CYTOMEGALOVIRUS/ or "HERPESVIRUS 3, HUMAN"/ or "HERPESVIRUS 4, HUMAN"/ or "HERPESVIRUS 1, HUMAN"/ or "HERPESVIRUS 2, HUMAN"/ or EBOLAVIRUS/ or MARBURGVIRUS/ or "CORONAVIRUS INFECTIONS"/ or "SEVERE ACUTE RESPIRATORY SYNDROME"/ or COVID-19/ or SARS-COV-2/	380783
6	4 or 5	696413
7	3 and 6	64979
8	("long covid" or "post covid" or postcovid).mp.	2457
9	7 or 8	66787
10	(therapy or therapies or managing or manage or management or manages or treat*).ti.	2495269
11	exp *THERAPEUTICS/	2635485
12	10 or 11	4623864
13	9 and 12	14770
14	limit 13 to randomized controlled trial	570

Table S2 - GRIPP-2 short form checklist [30].

Section and topic	Item	Description of involvement
1: Aim	Report the aim of PPI in the study	Patients and the public were involved in the development of the research question and design of the review.
2: Methods	Provide a clear description of the methods used for PPI in the study	The PPI activities were supported by patient and public involvement and engagement (PPIE) group and patient partner for the project (JC). We held an initial meeting with members of the PPIE group where members of the research team gave a presentation which highlighted the similarity between post-viral syndromes and Long COVID, the rationale for this review as well as our preliminary ideas. Following the presentation, there was a discussion of the review objectives. The PPIE group members were asked for their thoughts on the review objectives and its scope. The final review was shared with the PPIE group members for their thoughts and opinions. JC was also involved in the write up of the protocol and final manuscript.
3: Study results	Outcomes—Report the results of PPI in the study, including both positive and negative outcomes	During the design phase the PPIE group confirmed that the objectives were appropriate and comprehensive, they stressed the impact of Long COVID on employment and that the loss of income was very important to patients. JC also provided critical input and opinion during the write up of the protocol independently of PPIE group comments.<br>Patients and members of the public were not involved in the study selection, extraction, or synthesis phases.
4: Discussion and conclusions	Outcomes—Comment on the extent to which PPI influenced the study overall. Describe positive and negative effects	Therefore, a key goal of this work should be to identify potential non-pharmacological interventions that will facilitate the rehabilitation of patients with long COVID so that they continue to make progress and can adapt to new working methods. Based on their feedback, we ensured that this review reported interventions designed to improve the wellbeing of patients which will facilitate their return to work.
5: Reflections/critical perspective	Comment critically on the study, reflecting on the things that went well and those that did not, so others can learn from this experience	Early engagement with our PPIE group was critical to designing a research question with outcomes relevant to the patient population who will be most affected by any recommendations from this study.

Table S3 – Narrative description for risk of bias assessment

de Oliveira et al., 2019 [92]		
**Bias**	**Authors' judgement**	**Support for judgement**
Random sequence generation (selection bias)	Low risk	'Permuted block randomization was performed using the program Random Allocation 2.0'
Allocation concealment (selection bias)	Low risk	'Sealed, opaque, and sequentially numbered enveloped with a 1:1 allocation ratio'
Blinding of participants and personnel (performance bias)	High risk	Blinding of participants was not feasible for this study and therefore there may be risk of placebo effect etc.
Blinding of outcome assessment (detection bias)	Low risk	'Blinded assessor who did not know to which group each participant had been allocated'
Incomplete outcome data (attrition bias)	Unclear risk	All outcomes were reported on, but intention-to-treat (ITT) analysis was not carried out and it is unknown if this may have biased the results

Selective reporting (reporting bias)	Low risk	Outcomes were evaluated against the trial protocol and no discrepancies were found
Other bias	Low risk	No other sources of bias were identified
**Li et al., 2021 [90]**		
**Bias**	**Authors' judgement**	**Support for judgement**
Random sequence generation (selection bias)	Low risk	'Permuted allocation sequences for 1:1 block randomization (block size 10-14) stratified by hospital were computer-generated'
Allocation concealment (selection bias)	Low risk	'Allocation was concealed by central randomization and only revealed after baseline assessment through call to study center'
Blinding of participants and personnel (performance bias)	High risk	Blinding of participants was not feasible for this study and therefore there may be risk of placebo effect etc.
Blinding of outcome assessment (detection bias)	Low risk	'Patients and therapists were requested to not disclose allocation to assessors at any time during this study'
Incomplete outcome data (attrition bias)	Low risk	Intention-to-treat analysis was carried out in order to account for those lost to follow up and minimize loss of outcome data

Selective reporting (reporting bias)	Low risk	Outcomes were evaluated against the trial protocol and no discrepancies were found
Other bias	Low risk	No other sources of bias were identified
**Malik et al., 2020 [13]**		
**Bias**	**Authors' judgement**	**Support for judgement**
Random sequence generation (selection bias)	Low risk	'Participants were randomized to either mental training or care as usual in a 1:1 probability by a computer-based routine for block randomization'
Allocation concealment (selection bias)	Low risk	'Allocation concealment was ensured using sequentially numbered, opaque, sealed envelopes'
Blinding of participants and personnel (performance bias)	High risk	Blinding of participants was not feasible for this study and therefore there may be risk of placebo effect etc.
Blinding of outcome assessment (detection bias)	Unclear risk	States 'end-point evaluation was concealed from patients and therapists', but does not explicitly state whether the outcome assessors were themselves blinded or not

Incomplete outcome data (attrition bias)	Low risk	Intention-to-treat analysis was carried out in order to account for those lost to follow up and minimize loss of outcome data
Selective reporting (reporting bias)	Low risk	Outcomes were evaluated against the trial protocol and no discrepancies were found
Other bias	Low risk	No other sources of bias were identified
**Neumann et al., 2021 [91]**		
**Bias**	**Authors' judgement**	**Support for judgement**
Random sequence generation (selection bias)	Low risk	'One independent examiner performed bloc randomization using randomization.com'
Allocation concealment (selection bias)	Low risk	'Results were placed in dark sealed envelopes that were given directly to the examiner responsible for the intervention
Blinding of participants and personnel (performance bias)	High risk	Blinding of participants was not feasible for this study and therefore there may be risk of placebo effect etc.
Blinding of outcome assessment (detection bias)	Low risk	'One independent research assistant tabled and codified the data in order to blind the statistics'

Incomplete outcome data (attrition bias)	Low risk	Intention-to-treat analysis was carried out in order to account for those lost to follow up and minimize loss of outcome data
Selective reporting (reporting bias)	Low risk	Outcomes were evaluated against the trial protocol and no discrepancies were found
Other bias	Low risk	No other sources of bias were identified
**Silva-Filho et al., 2018 [93]**		
**Bias**	**Authors' judgement**	**Support for judgement**
Random sequence generation (selection bias)	Low risk	'A random numerical sequence was generated (www.randomization.com) to assign each participant'
Allocation concealment (selection bias)	Unclear risk	Unclear if or how allocation concealment was done
Blinding of participants and personnel (performance bias)	High risk	'Participants and researchers were blind to group allocation throughout the trial' 'Sham-tDCS was performed on 5 consecutive days with electrodes placed on the same position'

Blinding of outcome assessment (detection bias)	Low risk	'Participants and researchers were blind to group allocation throughout the trial'
Incomplete outcome data (attrition bias)	Low risk	Intention-to-treat analysis was carried out in order to account for those lost to follow up and minimize loss of outcome data
Selective reporting (reporting bias)	High risk	Did not report on one of the primary outcomes in the trial protocol (DN4 Questionnaire)
Other bias	High risk	'The City University of New York has patent on brain simulation with MB as the inventor. MB has equity in Soterix Medical Inc.'
